# Correction: Organizational and behavioral attributes’ roles in adopting cloud services: An empirical study in the healthcare industry

**DOI:** 10.1371/journal.pone.0320642

**Published:** 2025-03-20

**Authors:** The PLOS One Staff

## Notice of Republication

1. In the 2nd corresponding author name: Mohammed Al-Bsheish was changed to be “Mohammad Al-Bsheish.”
2. The abbreviation Mohammad Al-Bsheish’s affiliation Batterjee Medical College should be BMC, bot PMC.
3. Funding: The author(s) received no specific funding for this work. However, they declared that Publication was Funded by Universiti Kebangsaan Malaysia, under the grant DPB-2020-308.

## Supporting information

S1 File Originally published, uncorrected article. (PDF)

S2 File Republished, corrected article. (PDF)
