# Supplementary material for: Organizational and behavioral attributes’ roles in adopting cloud services: An empirical study in the healthcare industry
Source: PLoS One. 2023 Aug 25;18(8):e0290654. doi: 10.1371/journal.pone.0290654 (PMC10456173; doi:10.1371/journal.pone.0290654)
Supplement: S1 File — (DOCX) [file pone.0290654.s001.docx]

**Annexure A: Measurement scales**

| **Cost-effectiveness** | Adapted from Tehrani & Shirazi (2014) | |
| --- | --- | --- |
| CST1: Cloud computing decreases our capital expenditures. | | |
| CST2: Cloud computing decreases the investment in new infrastructure. | | |
| CST3: Cloud computing eliminates the cost of licensing new software. | | |
| CST4: Cloud computing eliminates the cost of upgrading the system. | | |
| CST5: Cloud computing decreases the cost of system maintenance. | | |
| CST6: Cloud computing decreases our IT costs (such as IT personnel)/ | | |
| CST7: Cloud computing decreases our operating costs. | | |
| **Hardware modularity** | Adapted from Tallon (2008) | |
| HM1: Software applications can be easily transported and used across multiple platforms. | | |
| HM2: Our user interfaces provide transparent access to all platforms and applications. | | |
| HM3: Our hospital offers multiple interfaces or entry points (e.g., web access) to external users. | | |
| HM4: Our hospital makes extensive use of middleware to integrate key enterprise applications. | | |
| **Software modularity** | Adapted from Tallon (2008) | |
| SM1: Reusable software modules are widely used throughout our systems development unit. | | |
| SM2: Legacy systems within our hospital do not hamper the development of new IT apps. | | |
| SM3: Functionality can be quickly added to critical applications based on end-user requests. | | |
| SM4: Our hospital can easily handle variations in data formats and standards. | | |
| **Internet network** | Adapted from Tallon (2008) | |
| IN1: Our hospital has a high degree of systems inter-connectivity | | |
| IN2: Our hospitals’ systems are sufficiently flexible to incorporate electronic links to external parties. | | |
| IN3: Remote users can seamlessly access centralized data. | | |
| IN4: Data is captured and made available to everyone in the hospital in real-time. | | |
| **Training availability** | Adapted from Igbaria et al. (1995) and Lin & Lee (2005) | |
| TR1: My hospital views employee training as an investment, not an expense. | | |
| TR2: My hospital provided extensive training in e-service systems. | | |
| TR3: Clarity of end-users’ role and objectives before training. | | |
| TR4: Availability of adequate course material during training. | | |
| TR5: Availability of IT support after training. | | |
| TR6: Training programs precede effective cloud service usage. | | |
| **Confirmation** | Adapted from Bhattacherjee (2001) | |
| CFT1: My experience using cloud services in the health information system was better than expected. | | |
| CFT2: The service level provided by cloud services in the health information system was better than I expected. | | |
| CFT3: Overall, most of my expectations from using cloud services in the health information system were confirmed. | | |
| **Behavioral control** | Adapted from Taylor and Todd (1995) | |
| BCT1: Using cloud services in health information systems was entirely within my control. | | |
| BCT2: I had the resources to use cloud services in the health information system. | | |
| BCT3: I had the knowledge to use cloud services in the health information system. | | |
| BCT4: I had the ability to use cloud services in the health information system. | | |
| BCP5: I would be able to use the cloud services in the health information system well for managing my work. | | |
| **Cloud Health Information System Utilization** | | Adapted from Davis (1989) |
| UTT1: I will use cloud services in the health information system on a regular basis in the future. | | |
| UTT2: I will frequently use cloud services in the health information system in the future. | | |
| UTT3: I will strongly recommend that others use it | | |

**Annexure B: Descriptive Statistics**

Table 1 Descriptive statistics of demographic factors

| **Demographic factors** |  | **Frequency** | **%** |  |
| --- | --- | --- | --- | --- |
| ***Gender*** |  | |  |  |
| Male |  | 95 | 90.5 |  |
| Female |  | 10 | 9.5 |  |
| ***Age (Years)*** |  | |  |  |
| 25-30 |  | 53 | 50.5 |  |
| 31-35 |  | 28 | 26.7 |  |
| 36-40 |  | 16 | 15.2 |  |
| >40 |  | 8 | 7.6 |  |
| ***Education*** |  |  |  | |
| Bachelor |  | 73 | 69.5 |  |
| High diploma |  | 12 | 11.4 |  |
| Master |  | 18 | 17.1 |  |
| PhD |  | 2 | 1.9 |  |
| ***Experience with the health information system? (Years)*** | | | |  |
| 1–2 |  | 55 | 52.4 |  |
| 2–4 |  | 31 | 29.5 |  |
| >4 |  | 19 | 18.1 |  |

| Item | Median | Mode |  |  |  |
| --- | --- | --- | --- | --- | --- |
| ***Cost Effectiveness (CST)*** | | | ***Training Availability (TR)*** | | |
| CST1 | 5.00 | 5 | TR1 | 4.00 | 5 |
| CST2 | 5.00 | 5 | TR2 | 4.00 | 5 |
| CST3 | 5.00 | 5 | TR3 | 4.00 | 5 |
| CST4 | 5.00 | 5 | TR4 | 4.00 | 5 |
| CST5 | 5.00 | 5 | TR5 | 4.00 | 5 |
| CST6 | 4.00 | 5 | TR6 | 4.00 | 4 |
| CST7 | 5.00 | 5 | ***Confirmation (CFT)*** | | |
| ***Hardware Modularity (HM)*** | | | ***CFT1*** | ***4.00*** | ***4*** |
| HM1 | 5.00 | 5 | CFT2 | ***4.00*** | 4 |
| HM2 | 5.00 | 5 | CFT3 | ***4.00*** | 5 |
| HM3 | 5.00 | 5 | ***Behavioral Control (BCT)*** | | |
| HM4 | 5.00 | 5 | BCT1 | 3.00 | 3 |
| ***Software Modularity (SM)*** | | | BCT2 | ***5.00*** | 5 |
| SM1 | 4.00 | 5 | BCT3 | 4.00 | 4 |
| SM2 | 5.00 | 5 | BCT4 | 4.00 | 4 |
| SM3 | 4.00 | 5 | BCT5 | 4.00 | 5 |
| SM4 | 4.00 | 5 | ***Utilization (UTT)*** | | |
| ***Internet Network (IN)*** | | | ***UTT1*** | ***4.00*** | 5 |
| IN1 | 5.00 | 5 | UTT2 | ***4.00*** | 4 |
| IN2 | 5.00 | 5 | UTT3 | ***4.00*** | 5 |
| IN3 | 5.00 | 5 |  |  |  |
| IN4 | 5.00 | 5 |  |  |  |

Table 2 Descriptive statistics for construct
