# Supplementary material for: Correction: Organizational and behavioral attributes’ roles in adopting cloud services: An empirical study in the healthcare industry
Source: PLoS One. 2025 Mar 20;20(3):e0320642. doi: 10.1371/journal.pone.0320642 (PMC11925271; doi:10.1371/journal.pone.0320642)
Supplement: S1 File — (PDF) [file pone.0320642.s001.pdf]

PLOS ONE

RESEARCH ARTICLE

Organizational and behavioral attributes' roles in adopting cloud services: An empirical study in the healthcare industry

Ahmed Meri[1]*, **Mohammad Khatim Hasan**[2], **Mohammed Dauwed**[3], **Mu'taman Jarrar**[4,5], **Ali Aldujaili**[6,7], **Mohammed Al-Bsheish**[8,9]*, **Salah Shehab**[10], **Haitham Mohsin Kareem**[11]

1 Department of Medical Instrumentation Techniques Engineering, Al-Hussain University College, Karbala, Iraq, **2** Faculty of Information Science and Technology, Universiti Kebangsaan Malaysia, Bangi, Selangor, Malaysia, **3** Computer Science, College of Science, University of Baghdad, Baghdad, Iraq, **4** Vice Deanship for Development and Community Partnership, College of Medicine, Imam Abdulrahman Bin Faisal University, Dammam, Saudi Arabia, **5** Medical Education Department, King Fahd Hospital of the University, Al-Khobar, Saudi Arabia, **6** Department Affairs of Student Accommodation, University of Baghdad, Baghdad, Iraq, **7** Department of Signal Theory and Communications, Information and Communication Technologies, University of Alcalá, Madrid, Spain, **8** Health Management Department, Batterjee Medical College (PMC), Jeddah, Saudi Arabia, **9** Al-Nadeem Governmental Hospital, Ministry of Health, Amman, Jordan, **10** College of Graduate Studies, Universiti Tenaga Nasional, Kajang, Selangor, Malaysia, **11** Department of Accounting, Southern Technical University, Basrah, Iraq

* dr.meri@huciraq.edu.iq (AM); mohammed.ghandour@bmc.edu.sa (MAB)

Abstract

The need for cloud services has been raised globally to provide a platform for healthcare providers to efficiently manage their citizens' health records and thus provide treatment remotely. In Iraq, the healthcare records of public hospitals are increasing progressively with poor digital management. While recent works indicate cloud computing as a platform for all sectors globally, a lack of empirical evidence demands a comprehensive investigation to identify the significant factors that influence the utilization of cloud health computing. Here we provide a cost-effective, modular, and computationally efficient model of utilizing cloud computing based on the organization theory and the theory of reasoned action perspectives. A total of 105 key informant data were further analyzed. The partial least square structural equation modeling was used for data analysis to explore the effect of organizational structure variables on healthcare information technicians' behaviors to utilize cloud services. Empirical results revealed that Internet networks, software modularity, hardware modularity, and training availability significantly influence information technicians' behavioral control and confirmation. Furthermore, these factors positively impacted their utilization of cloud systems, while behavioral control had no significant effect. The importance-performance map analysis further confirms that these factors exhibit high importance in shaping user utilization. Our findings can provide a comprehensive and unified guide to policymakers in the healthcare industry by focusing on the significant factors in organizational and behavioral contexts to engage health information technicians in the development and implementation phases.

Check for updates

OPEN ACCESS

Citation: Meri A, Hasan MK, Dauwed M, Jarrar M, Aldujaili A, Al-Bsheish M, et al. (2023) Organizational and behavioral attributes' roles in adopting cloud services: An empirical study in the healthcare industry. PLoS ONE 18(8): e0290654. https://doi.org/10.1371/journal.pone.0290654

Editor: Faisal Shafique Butt, COMSATS University Islamabad - Wah Campus, PAKISTAN

Received: January 10, 2023

Accepted: August 9, 2023

Published: August 25, 2023

Copyright: © 2023 Meri et al. This is an open access article distributed under the terms of the Creative Commons Attribution License, which permits unrestricted use, distribution, and reproduction in any medium, provided the original author and source are credited.

Data Availability Statement: All relevant data are within the paper and Supporting Information files.

Funding: The author(s) received no specific funding for this work.

Competing interests: The authors have declared that no competing interests exist.

I. Introduction

Health information system (HIS) refers to using Information Technology (IT) techniques to manage and retrieve records associated with individuals' health conditions or even the health sector activities. It is usually used to provide equitable, effective, and efficient healthcare services. Such computing health systems offer the availability for monitoring and following up on a patient's health status by healthcare providers. With this in mind, previous studies advised utilizing emerging technologies to improve the provided health services, allowing healthcare providers to detect, track, prevent, and treat disease [1]. Therefore, IT is considered the main antecedent in providing effective health services. The speeding up of innovative developments involving cloud computing services led to many implications in the healthcare era, such as involving managing and processing health records in distributed health environments [2]. Thus, cloud health applications can offer an exceptional advantage in optimizing health services. This is because of the dynamic settings of cloud computing that allow utility computing with its functionalities which is usually applied to manage health records in a distributed, ubiquitous, and pervasive way. Also, it supports multiple platforms, systems, and applications run at independent places. On top of that, cloud computing provides great features, including measured service, on-demand self-service, broad network access, rapid elasticity, and resource pooling. Furthermore, it assists in improving the current practices for delivering the service based on service types that can be mainly categorized into Software as a Service (SaaS), Platform as a Service (PaaS), and Infrastructure as a Service (IaaS).

Exploring the literature revealed that the SaaS is the most commonly used cloud service model by organizations for public and private health sectors. The PaaS models deliver a platform to customers to develop, run, and manage the applications without building and maintaining the infrastructure, while IaaS involves the platform infrastructure visualization. However, the current direction is to supply, apply, and process novel ways of providing services. There is a shortage of understanding regarding the low usage of cloud services [3].

Nowadays, many developing countries still suffer from big data management issues, especially in the health industry, making the operation more complicated [4]. Even though the use of cloud-based services has been raised in the healthcare industry, the implementation process is still in its infancy. The present health sector policies have highlighted the demands of new trend technologies to lower the socioeconomic disparities among healthcare organizations [5]. Yusof and Aziz [6] asserted that organizational readiness to change must be evaluated before information system adoption. Additionally, Mukred et al. [7] advised not to implement cloud computing without a research model that highlights the success factors for current application environments, in which a model must be applicable during emergencies such as pandemics [8]. Therefore, the key factors leading to successful cloud utilization must be identified. Despite a wide range of available literature on organizational structure and individual behavior constructs, very little research has addressed the empirical evidence among them [9]. As such, this research was motivated to model the relationships between the organizational structure enablers that affect individuals' behaviors to utilize cloud-based services in the public healthcare sector in Iraq, which will provide deep insight for policymakers and hospital managers of key success factors to enhance cloud HIS.

II. Background

Iraq is officially known as the Republic of Iraq, and the Ministry of Health operates its health care system, managing all public, military, and private hospitals. A health directorate controls each city in a hierarchical structure that manages all hospitals and primary health centers. The Ministry of Health has considered many attempts to adopt cloud computing models to

optimize their care practices by modifying the way of storing patients and other health records, thus permitting health professionals to efficiently manage and interpret patients' health conditions. In contrast, Al Hilfi et al. [10] highlighted numerous challenges with the current Iraqi healthcare system resulting from losing healthcare workers, especially physicians, besides the existing political interference. At the same time, they pointed out the need for efforts to provide healthcare facilities data to the ministries. Furthermore, Cetorelli & Shabila [11] asserted that the newly elected government (after the US invasion in 2003) should strengthen the healthcare sector and international contributors. Moreover, Hameed et al. [12] stated that challenges facing the health sector in Iraq to provide sufficient medical services relate to improper technology use, in which there is no defined procedure to share information.

The healthcare industry periodically improves its health information systems with the technology revolution worldwide. Cloud computing is seen as a prominent technology that offers a range of services to all sectors globally, especially the healthcare sector. The cloud HIS provides the required platforms for healthcare staff to communicate and share medical-related data across departments. The health systems are usually built on workflows [13] that include paper-based medical records, duplicated test results, and fragmented IT systems, to which most healthcare providers tend not to have access to the needed patient data when they must make a quick decision. Therefore, cloud service is an essential tool invented in developed and developing countries to alter health management practices [14]. Furthermore, Zhai et al. [15] acknowledged the use of IT services as an alternative way for healthcare professionals to offer health services and manage their patient's health status more efficiently.

Despite the most recent development of healthcare services, Iraqi healthcare facilities face some issues in accessing and delivering health-related data across departments, hospitals, and regions [16]. Healthcare institutions often face data management issues, especially with the limited availability, transfer, and recovery of medical data, all-pervasive access to medical documents, and IT resources. Therefore, Iraqi hospitals are confronted by large quantities of health data which are continuously increasing due to the growing number of patients and recent increased violence [10]. Thus, technological innovations such as utilizing cloud HIS can provide a reliable means to healthcare institutions.

Healthcare apps and health information can add value if organized and provisioned through cloud computing, where good storage capacities for medical sources are available. Furthermore, cloud computing can allow centrally stored medical data to be shared throughout departments. Based on these, cloud computing is believed to minimize IT-related costs by offering a structured HIS to all healthcare professionals. Utilizing cloud HISs is based on the technological expertise of users. Because physicians are always still far from technology, information technicians must guide the use of HISs and resolve technological issues. As such, [17] revealed that healthcare information technicians are key for successfully utilizing HISs in Iraq.

Few related studies were found in the literature that constructed cloud computing models in healthcare sectors. The researchers reviewed the previous models and their limitations to gain insight into cloud health systems utilization. They found that utilization and adoption models have been constructed to facilitate certain medical purposes in different countries. However, almost all studies on cloud healthcare services adoption imposed many limitations in terms of factors, use context, and reason for use, which are restricted to specific developed and developing countries.

Reviewing the models revealed that many studies examined some behavioral and/or organizational-related enablers according to the existing technology state implemented to satisfy particular purposes, with no study highlighting organizational exogenous factors' effects on an individual's behaviors to utilize the technology. As such, a real need exists for research to

improve health services by shedding light on the main concerns that may affect health information technicians' use of cloud services.

Consequently, this current research proposed organizational structure dimensions as a predictor exogenous variable that may affect the utilization of cloud computing services in the Iraqi public health sectors by intervening in health information technicians' behavioral aspects regarding control and confirmation. Moreover, the current study model construction was supported theoretically.

III. Model development and hypotheses

A. Underlying theories

The study model was constructed based on two theories; organizational theory [18] and the Theory of Reasoned Action (TRA) [19]. According to the literature, these theories describe the relationship between organizational structure and individual constructions for using cloud HIS. They define four main categories that affect the environment in terms of social structure, organization structure, culture, and technology. Hatch and Cunliffe (2013) stated that knowledge distributed throughout the organization can influence individuals' ability to work and the consequent outcomes. The outcome of a person is identified by environmental settings such as technology, physical structure, culture, and social structure. They highlighted the need to study any of the four aspects of organization theory contributing to an organization's problem. Hence, understanding the main parts can help describe the current technology usefulness and structure shortage within the Iraqi health sector to use cloud HIS. The theory assumes that promoting individuals' behavior is perceived by the associations amidst the technological structure and environment.

The association of organizational structure and transformations in someone's behavior can influence an individual's use of technology. Furthermore, organization theory can provide content for executive training programs. The theory describes the effects of communication on organizational perspectives to conduct successful managing or learning tools, design effective communication systems, or provide a suitable match of the network with the organization's needs. As a result, it is believed that a system deficiency may result in less coordination in Iraqi healthcare organizations. Moreover, scholars declared the importance of enrolling organizational theory in utilizing technology in the healthcare domain. For instance, Gao and Sunyaev [20] highlighted the need to understand the crucial attributes of this theory in formulating any technological innovation within the healthcare sector.

This study considered the link of the theory of reasoned action in describing how users' behavior can be influenced by their intentions to utilize or adapt technology. In the present study, TRA explains how healthcare IT personnel's behaviors can be influenced by their behavioral beliefs and cloud service adoption. The function of TRA is to regulate users' behavioral changes to create normative beliefs and the motivation to comply with the offered technology. Madden et al. [21] stated that an individual's positive behavior outcomes from a certain use could also make his behavior more positive towards performing that behavior. Positive behavior is, therefore, strongly associated with the amount of control an individual has that increases the required sense of motivation to meet the expectations of a task. The researchers, therefore, rely on the behavior-dependent variable as the main construct of the study's model outcomes.

Behavior is affected by behavioral beliefs and normative beliefs. In our case, behavioral beliefs reflect a user's attitude to confirm the use of the system, represented by the confirmation variable. Normative belief, multiplied by the motivation to comply with the offered services, directly affects a user's behavior for better control and, thus, successful adoption [22]. This is also evident in the healthcare sector, where Finlay et al. [23] found that healthcare

members' subjective norms influence their intention to perform health behavior. Therefore, in the present study, attitude is represented by confirmation, while subjective norm vs. motivation to comply as control belief is represented by control, which is assumed to influence healthcare professionals' behaviors to utilize cloud HIS.

B. Hypotheses development

Using the above-discussed theories, the researchers proposed the relationships between the research constructs. The relation of contexts of this research has been linked to each factor as defined in the following subsections.

1) Organizational structure domain. The present study investigates the essential role of organizational structure in impacting individual utilization and adaptation of technology within the organizational environment. An organizational structure is presented by establishing the foundation upon which organization functions are executed to accomplish desired aims. Furthermore, the pertinent aspects of organizational structure have previously been proven to impact individual behaviors, to regulate performance and adaptation capabilities [24]. Wang and Lee [25] proposed that various factors must be considered to appropriately utilize technology dependent on the outcome of prior experiences. Additionally, these factors should appropriately satisfy the fundamental prerequisites for technological utilization. Thus, the present study emphasizes certain elements within the organizational structure responsible for executing health system-based cloud in the context of prominent Iraqi hospitals. The belief is commonly held that healthcare members perceive organizational structure as a key influencer of behavior.

To augment this belief, the organizational structure is perceived as a sufficiently crucial factor that may lead to difficulties in operation for healthcare members. As such, the purpose of organizational elements is to optimize the impact of individual factors, especially when utilizing technology that benefits organizational operations [9]. Thus, in the present study, we considered the crucial function of organizational structure within the hospital environment. Furthermore, the subunit levels of behavior, confirmation, and control were also considered for the health information system-based cloud.

Prior literature has already covered organizational structure and its impact on individual behavior [26]. However, there is a lack of literature in studies focusing on organizational structure elements considered for health cloud computing technology. Therefore, the researchers assumed that organizational structural factors in terms of cost-effectiveness, hardware modularity, software modularity, Internet network, and the provision of training might enhance ITs' confirmation and behavioral control to utilize cloud health information systems in the Iraqi health sector. The following sub-sections demonstrate each factor and its relation to the study context.

• Cost-effectiveness

Cost-effectiveness is a term employed to evaluate emerging new technologies by comparing the financial aspects of such technologies in a side-by-side comparison [27]. The literature shows that cost-effectiveness is a re-occurring factor referred to as a crucial element in technology utilization and adoption. The partial integration of technology in an organizational context is expensive [28], implying that the perceived cost of cloud computing services for HISs will benefit confirmation and control for utilization. Ling et al. [29] emphasized the significance of cost-effectiveness for the effective adoption of advanced services, which depended on the apparent deficiency of cheap technological alternatives.

Previous studies have considered the prominent role of cost-effectiveness in technological utilization and adoption within the organizational setting. Lena et al. [30] conducted a scoping review for cloud computing utilization in the healthcare sector. Their study asserted that one benefit of the cloud is cost reduction, which many case studies in different developed and developing countries support. Consequently, in the present study, we considered the importance of cost-effectiveness for regulating healthcare information technicians' confirmation and behavioral control for adopting the cloud HIS and thus, the following hypothesizes were posited:

$H1_a$: Cost-effectiveness of cloud HIS positively influences healthcare information technicians' confirmation of the technology.

$H1_b$: Cost-effectiveness of cloud HIS positively influences healthcare information technician's behavioral control of the technology.

• Hardware Modularity

Hardware modularity or compatibility is an organizational element popularly brought up for its impact on technological utilization and adoption. Byrd & Douglas [31] emphasized the significance of facilitating non-rigid IT infrastructure for adapting to organizations' ever-changing goals and requirements. Improving hardware modularity by facilitating the required equipment and machinery responsible for developing and debugging health-related issues between departments has previously been shown to benefit the scalability of healthcare applications [32]. Thus, healthcare members must be provided with enough hardware to effectively contribute and heighten their confirmation of effectively managing and transferring medical data among different departments in the hospital. Hsu & Lin [33] emphasized that perceived user confirmation of service usage is a crucial factor responsible for environmental components leading to user desire to keep employing the particular technology tool. Susanto et al. [34] explored this notion, in which the role of organizational structure was discussed. It was said that the organizational structure comprised available resources, which promoted user confirmation to keep utilizing technology for long-term usage. Previous studies have also examined the effects of hardware modularity state to facilitate the required user control for task management and performance purposes. The literature showed that perceived control of environmental elements is a factor popularly employed for forming relevant theories of control, some of which include the locus of control, self-efficacy, and self-competence theories [35]. Based on this, the following hypotheses were posited:

$H2_a$: Hardware modularity for running a cloud HIS positively influences healthcare information technicians' confirmation of the technology.

$H2_b$: Hardware modularity for running a cloud HIS positively influences healthcare information technicians' behavioral control of the technology.

• Software Modularity

It is a crucial factor for evaluating the connectivity reduction among program parts, thus reducing the complexity of the code structure. A relevant notion to consider is that the design modularity for process development and comprehensibility comprises the execution of multiple system tasks [36]. For instance, Sant'Anna et al. [37] stated the importance of software modularity in enabling individuals to utilize and adopt the technology. Software modularity represents the standardization of units meant to be as flexible as possible for utilization. Prior studies have illustrated the association between software modularity and individual utilization behavior in relation to the complexity and capacity of available resources [38]. Sun et al. [39] highlighted the importance of up-to-date software for supporting organizations to upkeep

efficient technology utilization, depending on IT infrastructure flexibility, thus proving how applications may be restructured quite easily. Moreover, Green et al. [40] connected concepts of software modularity, including quality, productivity, and other such repeatable processes, for changing individuals' control over the pertinent processes.

Based on the discussion, the argument could be made that software strongly affects individual behavior to employ cloud HIS. To assess this, the following hypotheses were posited:

$H3_a$: Software modularity for running a cloud HIS positively influences healthcare information technicians' confirmation of the technology.

$H3_b$: Software modularity for running a cloud HIS positively influences healthcare information technicians' behavioral control of the technology.

• Internet Network

Internet network is another important factor responsible for shaping networks within the organizational setting. In the present study, a network is defined as a computer service facilitating data exchange and information among places where the Internet is the main data exchange channel over the cloud. The availability and capability of the Internet enable people to manage and modify data through various channels. The study assumptions were substantiated by Steinbart & Nath [41], who proposed that a network is a key element responsible for organizational management. Tshimula et al. [42] also said that a network might positively affect the user engagement experience owing to its role in forming utilization decisions in the IT sector. Furthermore, network facilitation in the IT sector is mainly investigated to provide users with the means to achieve heightened workplace efficiency.

The extant literature has highlighted that developing countries face Internet issues related to poor telecommunication capabilities and power supplies that result in insufficient bandwidths and low connectivity [43]. These issues and network infrastructure deficiencies among government organizations, individuals, and the technology market within these developing countries suggest that utilizing cutting-edge technologies in developing countries is challenging. Consequently, the effectiveness of cloud-based HISs in Iraq could depend on technology infrastructures in which poor network telecommunication infrastructure limits healthcare members' access and exchange of inter-departmental medical data [44], thus, less utilization of cloud HIS. Based on the above, the following hypotheses were posited:

$H4_a$: Internet Network for using a cloud HIS positively influences healthcare information technician's confirmation of the technology.

$H4_b$: Internet Network for using a cloud HIS positively influences healthcare information technician's behavioral control of the technology.

• Training Availability

Training is the available resource by which organizations strive to facilitate individuals with the means to garner knowledge necessary for technology operation [45]. Past studies have shown that individual members of an organization having access to tools for learning technical details require an organization's adoption of innovation strategies. Prior studies have constantly highlighted the importance and availability of training for driving one's decision for technology adoption and that training could impact his/her perceptions about utilizing a technology. For example, Ghaleb et al. [46] highlighted that the availability of appropriate training for practitioners was compulsory for strong telehealth adoption. However, they asserted that the availability of telehealth-related training for practitioners was not understood yet. Bello et al. [47] stated that the deficiency of structured training, as well as computer accessibility, contributed to poor IT knowledge, especially among healthcare professionals. Therefore,

training could be considered a key driver for cloud HIS utilization, whereby training could facilitate the effective utilization of telehealth members, such as patients, physicians, and IT personnel. Additionally, training quality is expected to benefit healthcare members' perceived control of the technology [48]. Lee [49] mentioned that the deficiency of training resources would negatively influence user confirmation as to their control over technological utilization. Therefore, the following hypotheses were posited:

$H5_a$: Training availability to use a cloud HIS positively influences healthcare information technician's confirmation of the technology.

$H5_b$: Training availability to use a cloud HIS positively influences healthcare information technician's behavioral control of the technology.

2) Individuals domain. Demographics, perceptions, and behaviors are the major perspectives for studying the characteristics of individuals [50]. In the present study, the domain of individuals is a main factor that may affect individuals' behaviors in adopting and utilizing technology. In healthcare, individual factors are key enablers for individuals' beliefs [51]. However, healthcare information technician's belief impact on the adoption of cloud HIS has not yet been studied. Hossain et al. [44] emphasized that individuals' positive perceptions of maintaining a specific user behavior will establish a responsibility feeling that makes them much more committed with more confirmation and control behavior to do their tasks. In light of that, healthcare ITs in the Iraqi healthcare sector may have positive attitudes toward executing specific behavior in their hospitals. As a result, researchers in the present study hypothesized the expected relation of healthcare ITs' confirmation and control to positively impact the usefulness of cloud HIS in Iraqi health settings.

• Confirmation

According to Bhattacherjee [52], confirmation refers to the perception of congruence between expectation to utilize novel technology and the real performance achieved. As such, it is a necessary part of an individual's cognitive belief based on past experience using the technology. It has been approved that expectation confirmation can forecast individuals' expectations to employ a specific technology [53]. Furthermore, Lu et al. [54] asserted that individuals' confirmation of a certain technology is based on their motivation to utilize it further by considering several environmental and personal traits. Findik-Coşkunçay et al. [55], who found that certain service users usually adopt this technology, support this notion.

Moreover, previous literature asserted that individuals' behavioral confirmation of certain technology is connected to his/her usage of this technology [56]. As a result, confirmation was found to alter users' expectations of utilizing technology in their workplace. Thus, researchers in the present study considered the important role of confirmation in facilitating the cloud HIS utilization and posited the following hypothesis:

H6: Confirmation of the cloud HIS positively influences healthcare information technicians' utilization of such technology.

• Behavioral control

Behavioral control is connected to the term "self-efficacy," which can be defined as "the reflection of users' confidence in the ability to exert control over his/her own motivation, behavior, and social environment" [57]. A user's belief is usually related to behavioral control, in which individuals may have normative expectations towards technology utilization to establish their normative beliefs facilitating an improved control [58]. According to Iskandar et al. [59], the effective utilization of technology entails that an organization ensures that users possess sufficient control because insufficient control would lead to user dissatisfaction and reduced

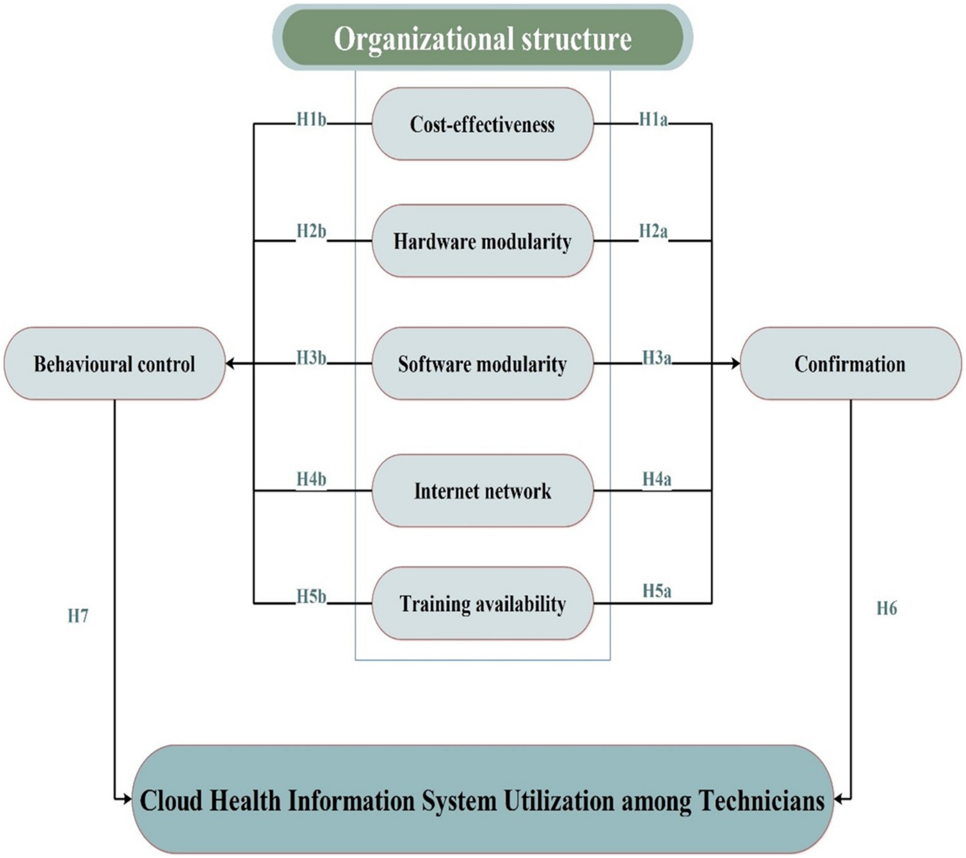

Fig 1. The study proposed model and hypotheses.

https://doi.org/10.1371/journal.pone.0290654.g001

system utilization. Thus, organizations must facilitate instruction to teach users how to properly control the system.

Cloud HIS is a complex operation for users related to the external control that a cloud vendor provides. Thus, executing actions for a particular situation may be affected by the external control locus of the cloud, which applies extraneous factors [60]. Therefore, researchers in this study were motivated to include behavioral control of cloud systems as an intervening variable that may affect users' utilization of cloud technology based on the organizational structure enablers. Consequently, the following hypothesis was posited:

H7: Behavioral control towards the cloud HIS positively influences healthcare information technicians' utilization of such technology.

As a result, Fig 1 shows the study proposed model and hypotheses.

IV. Methodology

A. Research design

This study employed a cross-sectional quantitative research approach to validate the proposed model. The unit of analysis was at the individual level. Neuman & Kreuger [61] expressed that the survey method will be suitable for studying research objectives or questions dealing with behaviors or beliefs. Consistent with this point of view, Zikmund [62] stated that surveys are significantly better methods for measuring opinions, awareness, and behavior. As such, the questionnaire survey technique was used to explore information technicians' views about the

research variables. A questionnaire is an appropriate data collection tool to investigate the relationships between variables across a large population for verification and validation purposes [63].

B. Sampling and data collection

The target population of this research was restricted to healthcare facilities operating in Baghdad Governorate, Iraq (the capital). This selection of these facilities was based on the availability of cloud computing, as the capital city healthcare sector receives much more intention to utilize it than other cities in Iraq. Of the thirty-nine public hospitals in Baghdad, only four general hospitals participated in this research study (Al-Karkh General Hospital, Baghdad Teaching Hospital, Al Kindi General Teaching Hospital, and Al-Yarmouk General Teaching Hospital). These hospitals were recommended by the director of state healthcare in Baghdad based on the number of IT personnel, medical cases, beds, physicians, and patients. Furthermore, these hospitals were selected due to their capacity to offer various health aids.

Information technology technicians were chosen as the population to sample because they could provide the needed information and are the backbone of the healthcare industry information systems [64]. Typically, they are responsible for technical activities that may influence the healthcare staff's control over systems. This approach aligns with Ratnam et al. [65], Harfoushi et al. [66], and Alharbi et al. [67], who investigated healthcare technicians' perceptions of the adoption of cloud computing services in the health sector through organizational technology factors. Thus, this study considered the role of information technicians' perspectives in formulating the utilization of cloud HISs in Iraq.

Furthermore, this research applied a simple random sampling technique, which gives generalizability and flexibility in selecting respondents randomly from the targeted population. Moreover, this sampling technique has a lower bias than other techniques [68]. One hundred and forty-six information technicians aware of cloud services were identified in the targeted hospitals.

An online questionnaire link was administrated to collect their views regarding the research variables. The online questionnaire was accessible for three months, and 109 respondents returned the questionnaire, which was considered adequate [69, 70], as the G*Power software showed a minimum required sample size of 74 respondents. The initial response rate was 74.65%, but 105 returned questionnaires were usable. Thus, the effective response rate was 71.9%, which is considered acceptable.

C. Instrument development

The instrument comprised five parts. The first part described the research, its significance, and the objectives and assured confidentiality to the participants. The second part asked demographic questions to gain more insights regarding the respondents' background related to their gender, age group, education level, and experience using cloud health systems. The last three parts of the questionnaire were constructed based on the contents of each construct, using a 5-Likert scale, in which participants provided an exact response for each item. The scale ranged from 1 = xxx, 2 = xxx, 3 = xxx, 4 = xxx, and 5 = xxx. Adding "Neutral" as an option can reduce the bias in data collection as respondents are not forced to choose a positive or negative answer [71].

The instrument was developed by adapting items from various studies (see Annexure A in S1 File: Measurement scales). Cost-effectiveness items were adapted from Tehrani & Shirazi [72], hardware modularity, software modularity, and internet network items were adapted from Tallon [73], items measuring training availability were adapted from Igbaria et al. [74]

and Lin and Lee [75]. Taylor and Todd's [76] items measuring respondents' control over cloud computing services in HIS were adapted for individual-related variables. Meanwhile, items for respondents' confirmation of the services were adapted from Bhattacherjee [52]. Finally, respondents' views about cloud computing service utilization were adapted from Davis [77].

Before collecting data, the survey instrument was examined for reliability and validity. Five specialists in information science, cloud computing, e-health, social sciences, HIS adoption, and one IT technician working in a hospital reviewed the questionnaire for content and face validity. This review helped determine the questionnaire's validity, clarity, understandability, and completion. Based on their feedback, the instrument was modified slightly.

Moreover, a pilot study was performed using snowball sampling to explore the questionnaire's reliability and confirm that it was free of errors and fit the Iraqi health context. Ten information technicians with experience in using cloud HIS were invited to participate, and 6 of those 10 invited their colleagues to participate. Finally, 32 healthcare information technicians participated in the pilot study. The results revealed that reliability was achieved, as Cronbach's alpha scores ranged between 0.696–0.848. Moreover, before distributing the online questionnaire, it was back-translated by two independent translators from English into Arabic to check for errors. The final Arabic-language questionnaire included a guide for answering the survey, and all items and constructs were described in detail to ensure that all respondents understood what they were answering.

D. Data analysis

Three main phases were performed to analyze data: data cleaning, sample descriptive statistics, and PLS-SEM (partial least square structural equation modeling) analyses. First, researchers checked whether the received data were clean and ready to be analyzed by looking for missing data (using the multiple imputation feature to analyze the patterns and impute the missing data values), outliers (using boxplots), normality distribution (using skewness and kurtosis tests), and collinearity (using variance inflation factors test). Before proceeding to analyze data using PLS-SEM, descriptive statistics analysis was performed; this analysis involved describing the demographic and construct variables based on the received data, in which the demographic information was described via measuring frequencies while descriptive statistics of the constructs were measured via the central tendencies. Because the received data were ordinal, non-parametric tests were carried out in which mode and median tests were used to determine the central tendencies for each item [68, 78].

Finally, the data analyses were performed using PLS-SEM, a non-parametric second-generation multivariate software, to examine the instrument's psychometric properties and the hypotheses statistically. Smart-PLS version 3.0 [79] was the software for statistical analyses. A systematic analysis was followed according to the recommendations of Hair et al. [70]. The reflective measurement model was evaluated based on two tests, convergent validity and discriminant validity. Convergent validity tests the internal consistency reliability by examining the composite reliability (CR), Cronbach's alpha coefficient, and the average variance extracted (AVE) at the construct's level. Additionally, the convergent validity test examined the individual indicator reliability and communality for the indicator's level. Second, discriminant validity was examined by testing the cross-loadings, Fornell-Larcker criterion, and Heterotrait-Monotrait (HTMT). After confirming the measurement model validity, the study followed a systematic assessment of the structural model according to the recommendations of Hair et al. [70], which comprised several steps: 1) path coefficient; 2) coefficient of determination (R^2); 3) effect size (f^2); 4) predictive relevance (Q^2); and 5) importance-performance map analysis (IPMA).

V. Findings

A. Data preparation

The data cleaning and screening phase were conducted before analyzing the data. Checking for missing data resulted in no missing data with the returned questionnaires, as all questions were required in the online distributed version. Four responses that resulted in extreme outliers were deleted. Skewness and kurtosis tests showed that the standard error score for skewness was 0.236, and for kurtosis was 0.467. In addition, constructs' skewness values ranged (from -2.183 to -0.986), while kurtosis values ranged (from -0.335 to 5.274). These results indicated that data were normally distributed. Lastly, a collinearity test using the VIF (Variance Inflation Factors) test revealed no critical issue with multicollinearity for all variables in which the inner VIF values ranged between 1.454 and 3.665.

B. Descriptive analysis

Four key demographic characteristics were gathered: age, gender, educational level, and experience using cloud computing services. These characteristics were included to provide an in-depth understanding of the sample and their appropriateness to respond to the study's survey. Annexure B in S1 File presents the descriptive statistics for all constructs that show the frequency and percentage for each demographic construct and the central tendencies. The majority highly agreed upon the proposed relationships of all exogenous endogenous variables. Moreover, most results were found to potentially influence information technicians' confirmation and control of cloud computing services. This result may imply that technicians' confirmation and behavioral control of cloud computing was mostly associated with the facilities offered by the hospital and cloud provider. Furthermore, we found a high agreement among all information technicians toward utilizing cloud services. The majority would recommend cloud computing to other healthcare members. Considering these observations, using cloud computing was positively perceived for its role in helping to manage health information within and between departments and hospitals.

C. Measurement model

The evaluation of the model involves testing convergent and discriminant validity. As for the convergent validity assessment, results illustrated in Table 1 showed that in terms of item level, the loadings for all indicators were greater than 0.7, and the commonality for all items exceeded the threshold of 0.5. For the construct's level, AVE, Cronbach's alpha, and composite reliability also exceeded the acceptable thresholds of 0.7, 0.7, and 0.5, respectively. These results confirm convergent validity [70]. Subsequently, Tables 2 and 3 present the Fornell-Larcker and HTMT results; all constructs' square roots of AVEs were greater than the correlation of this construct with other constructs in the model. Likewise, all the HTMT values were within the acceptable threshold of 0.85 [80]. Moreover, the cross-loading test shows that all indicators were highly loaded on their respective constructs. As such, discriminant validity was met; thus, the validity of the measurement model was confirmed.

D. Structural model assessment

1) Path coefficient. A bootstrapping procedure with 5000 resamples assessed the significance level of each path in the structural model [81]. Table 4 shows the bootstrapping path coefficient results regarding β, t-statistics, and p values. Considering a t-statistics threshold of 1.65 for one-tailed tests at a p-value of 5% resulted in three rejected hypotheses. Cost-effectiveness was not associated with confirmation (β = 0, t- statistics = 0.002, p-value = 0.499) or

Table 1. Measurement model assessment–Convergent validity.

Constructs	Items	Construct Level			Indicator Level	
		Cronbach's alpha	Composite Reliability	AVE	Loading	Communality
Cost-effectiveness (CST)	CST1	0.966	0.972	0.832	0.930	0.864
	CST2				0.928	0.861
	CST3				0.886	0.784
	CST4				0.908	0.824
	CST5				0.904	0.817
	CST6				0.928	0.861
	CST7				0.899	0.808
Hardware Modularity (HM)	HM1	0.963	0.973	0.900	0.939	0.881
	HM2				0.932	0.868
	HM3				0.968	0.937
	HM4				0.956	0.913
Software Modularity (SM)	SM1	0.968	0.976	0.911	0.949	0.900
	SM2				0.965	0.931
	SM3				0.951	0.904
	SM4				0.952	0.906
Internet Network (IN)	IN1	0.906	0.934	0.787	0.924	0.853
	IN2				0.845	0.714
	IN3				0.865	0.748
	IN4				0.899	0.808
Training Availability (TR)	TR1	0.913	0.932	0.697	0.859	0.737
	TR2				0.845	0.714
	TR3				0.838	0.702
	TR4				0.819	0.670
	TR5				0.834	0.695
	TR6				0.812	0.659
Confirmation (CFT)	CFT1	0.822	0.894	0.738	0.878	0.770
	CFT2				0.839	0.703
	CFT3				0.860	0.739
Behavioral Control (BCT)	BCT1	0.890	0.919	0.695	0.785	0.616
	BCT2				0.846	0.715
	BCT3				0.842	0.708
	BCT4				0.834	0.695
	BCT5				0.860	0.739
Utilization (UTT)	UTT1	0.793	0.877	0.705	0.846	0.715
	UTT2				0.832	0.692
	UTT3				0.841	0.707

https://doi.org/10.1371/journal.pone.0290654.t001

behavioral control (β = 0.004, t- statistics = 0.049, p-value = 0.480), while we found that behavioral control did not affect the utilization of cloud health information system (β = 0.199, t- statistics = 1.188, p-value = 0.118). All other hypotheses were statistically accepted. Furthermore, the researchers reported the 95% confidence intervals for each path in the structural model that can help estimate coefficient stability in which the hypothesis rejected of path equal zero for all proposed paths and a significant effect is assumed.

2) Coefficient of determination, effect size, and predictive relevance. The model's predictive power (R^2) shows that the overall power account for 71%, 65%, and 31% of confirmation, behavioral control, and cloud HIS utilization, respectively (see Table 5). This result

Table 2. Fornell-Larker criterion results.

	BCT	CFT	CST	HM	IN	SM	TR	UTT
BCT	0.834							
CFT	0.669	0.859						
CST	0.357	0.339	0.912					
HM	0.749	0.784	0.420	0.949				
IN	0.688	0.762	0.193	0.754	0.884			
SM	0.730	0.736	0.498	0.753	0.645	0.955		
TR	0.666	0.659	0.331	0.671	0.589	0.621	0.835	
UTT	0.477	0.549	0.376	0.497	0.519	0.401	0.477	0.840

https://doi.org/10.1371/journal.pone.0290654.t002

indicates that the confirmation had a high effect, behavioral control had a moderately high effect, but the utilization construct was rather weak but still acceptable [81]. Furthermore, results showed that all exogenous variables influenced their corresponding endogenous variables with an acceptable effect size (f^2) to which the path is connected [82], except for the cost-effectiveness that did not affect confirmation or control, as both paths were already insignificant. Moreover, researchers tested the model's out-of-sample predictive power using Stone-Geisser's Q^2 value [83, 84] by using the blindfolding process [85]. The results confirm the predictive relevance of all dependent variables in the structural model. According to Hair et al. [70], the explanatory strength and predictive significance confirm the model's predictive validity.

3) Importance-Performance Map Analysis (IPMA). The IPMA was used to generate information on the constructs' importance to explain other dependent constructs in the structural path model and take into account the performance of those constructs. IPMA contrasts the total effects (importance) of the structural model with the average scores of the exogenous variables (performance) to highlight significant areas of enhancement for management activities or perhaps the particular focus of the model [70]. The exogenous variables that gain a strong total effect perform well for their corresponding endogenous variables. However, low performance was observed with low average scores of exogenous variables. Thus, the researchers performed the IPMA procedure using Smart-PLS 3.0.

The results tabulated in Table 6 show that the internet network was the most important factor shaping the confirmation of individuals (32.7%), while cost-effectiveness had no effect (zero importance) on their confirmation with a high performance (81.4). Also, software modularity had high importance (24%), with relatively lower performance than other constructs (71.9). The IPMA results for the behavior control dependent variable revealed that the software modularity was the most important construct shaping an individual's behavioral control

Table 3. HTMT results.

	BCT	CFT	CST	HM	IN	SM	TR	UTT
BCT								
CFT	0.779							
CST	0.373	0.368						
HM	0.807	0.881	0.429					
IN	0.763	0.881	0.199	0.806				
SM	0.782	0.824	0.508	0.78	0.686			
TR	0.732	0.758	0.34	0.713	0.641	0.657		
UTT	0.549	0.669	0.42	0.556	0.602	0.446	0.544	

https://doi.org/10.1371/journal.pone.0290654.t003

Table 4. Bootstrapping path coefficients.

Hypotheses		Path coefficients (β)	t- values	p-values	95% Confidence Intervals	
					Lower level	Upper level
H1a	CST -> CFT	0000	**0.002**	**0.499**	-0.111	0.444
H1b	CST -> BCT	0.004	**0.049**	**0.480**	-0.218	0.657
H2a	HM -> CFT	0.262	1.828	0.034	0.135	0.147
H2b	HM -> BCT	0.241	1.657	0.049	0.164	0.170
H3a	SM -> CFT	0.239	2.177	0.015	0.012	0.471
H3b	SM -> BCT	0.292	2.146	0.016	0.042	0.509
H4a	IN -> CFT	0.327	3.577	<0.001	0.027	0.367
H4b	IN -> BCT	0.195	1.857	0.032	0.177	0.470
H5a	TR -> CFT	0.143	1.695	0.045	0.075	0.515
H5b	TR -> BCT	0.207	2.247	0.012	0.053	0.409
H6	CFT -> UTT	0.416	3.126	0.001	0.070	0.379
H7	BCT -> UTT	0.199	**1.188**	**0.118**	-0.005	0.273

https://doi.org/10.1371/journal.pone.0290654.t004

(29.2%) with a relatively low performance compared to other constructs (71.9%), in which the cost-effectiveness gained a higher performance (81.4%) with zero importance, as it had no significant effect on the behavioral control. As for the cloud HIS utilization construct, we can see that the hardware modularity was the most important construct (41.6%) in shaping the utilization of cloud HIS from respondent's perceptions, with relatively high performance compared to other constructs (78.6%), in which the software modularity had the most high performance among other constructs (81.4%).

VI. Discussion

Drawing on the statistical analysis findings, it is evident that hardware modularity, software modularity, Internet network, and training availability positively influenced information technicians' confirmation and behavioral control. However, only cost-effectiveness did not influence confirmation and behavioral control. Then, the researchers examined how the last affected their utilization of cloud computing services and found that only confirmation positively influenced this utilization.

The results revealed that the cost-effectiveness of cloud services had no significant associations with confirmation and behavioral control when considering using it in the workplace. The assertion driven by this relationship was that whether there is a certain cost allocation, information technicians' expectations of cloud effectiveness in the Iraqi hospitals will not be

Table 5. f^2, R^2, and Q^2.

Construct	f^2			R^2	Q^2
	BCT	CFT	UTT		
CST	0	0			
HM	0.048	0.068			
IN	0.043	0.146			
SM	0.091	0.073			
TR	0.066	0.038			
BCT			0.032	0.650	0.429
CFT			0.141	0.711	0.484
UTT				0.310	0.206

https://doi.org/10.1371/journal.pone.0290654.t005

Table 6. Importance-Performance matrix.

Exogenous LV	Confirmation (CFT)		Behavioral control (BCT)		Utilization (UTT)	
	Performance	Importance	Performance	Importance	Performance	Importance
CST	**81.428**	0	**81.428**	0.004	73.355	0.199
HM	78.639	0.262	78.639	0.241	79.362	**0.416**
SM	71.963	0.239	71.963	**0.292**	**81.428**	0.001
IN	81.334	**0.327**	81.334	0.195	78.639	0.157
TR	73.212	0.143	73.212	0.207	71.963	0.158
CFT					81.334	0.175
BCT					73.212	0.101

https://doi.org/10.1371/journal.pone.0290654.t006

affected. This conclusion can be explained by the fact that the expenditure by the health institution may not necessarily impose technical limitations to run and use cloud services. Torre-Díez et al. [86] support this. They claimed that little cost-utility and cost-effectiveness evidence exists for e-health systems concerning the use of telemedicine for reducing costs, but not all. However, this result contrasts with Henderson et al. [87], who warned policymakers not to put the lack of cost-effectiveness as evidence to avoid or neglect technology utilization. It is also assumed that Iraqi hospitals can still invest more resources to upgrade cloud services to involve multiple users and platforms. By doing so, information technicians may perceive the potential of cost-effectiveness in forming their behavioral confirmation and control about the cloud services.

Results showed that hardware modularity significantly and positively impacted their confirmation and behavioral control when they used cloud systems. These results align with the work of Saleem et al. [88], who asserted the importance of hardware elements in driving an individual's behavior to use technology. Moreover, this finding is consistent with Ahmadi et al. [89], who highlighted that the hardware factor could affect the implementation of a hospital's information system. Additionally, information technicians' expectations towards the current hardware modularity may need to be further expanded to comply with modern health protocols and communication demands between and within hospitals. Furthermore, software modularity positively impacted intervening factors to utilize cloud services. Thus, software has an important role in reducing each piece of an application that must be transferred from the server to the client [90]. This finding supports the works of Iacona et al. [91] and Al-Sakran [92], who emphasized the role of software modularity in empowering the overall management practices of health data which potentially could give rise to the confirmation of an individual in a certain context. In addition, the results may provide new insights into the current understanding of software modularity in developing countries' healthcare settings, which has been less examined.

Additionally, the Internet network significantly and positively impacted the information technicians' confirmation and behavioral control of the cloud health system. This result aligns line Finley et al. [93], who addressed the direct impact of network quality on users' technology behavior. The behavior of users might vary from one to another based on the levels of Internet network quality; thus, accounting for these differences may eventually drive their confirmation of the technology to their settings. Furthermore, training availability was found to be significant on both intervening variables. Thus, the lack of training in the healthcare sector may relatively influence persons' expectations of the technology. Healthcare professionals who receive less training support may not fully perceive the benefit of certain technologies in different medical settings [94]. This study's findings extend the work of Nkuma-Udah et al. [95] on how low training quality could negatively influence individuals' use of health systems. Additionally,

previous scholars frequently addressed the training availability aspect associated with the individual's behavioral control and expectations [96].

Confirmation was found to positively influence health information technicians' utilization of cloud technology. IT personnel have to be equipped with modern tools and technological advances in order for them to effectively perform their job [97]. Khayer et al. [53] extended this assumption, confirming that users' expectation confirmation has an essential role in the successful adoption of cloud computing. Commonly, users are required to be introduced to basic health management skills and ensure providing control concerning the current trends in the information technology revolution.

Finally, the result of testing H7 was not significant. This can be explained by the fact that information technicians have the knowledge required to easily use and adapt cloud systems within healthcare settings. Guo et al. [98] explained that the effects of perceived behavioral control in a healthcare professional context may not warrant its decomposition. The resulting behavior from using a certain system may not be necessary in line with the overall usage experience. Therefore, although no relationship was found between behavioral control and utilization of the cloud, many potential reasons still need to be explored to address ways and practical strategies for increasing information technicians' control with modern technological tools.

Extending the discussion of our findings by the importance-performance map analysis (IPMA) was very informative. Internet network is the main important construct in formulating individuals' confirmation. Scholars may focus more on improving the performance of Internet networks and hardware modularity to improve the overall performance of users' confirmation to be willing and motivated to utilize cloud systems. Also, more research is needed to improve the performance of software modularity in health organizations to improve users' confirmation. Moreover, behavioral control is mainly formulated by the most important construct of software modularity, even if it gained a relatively low performance, but future research could focus on improving its performance besides hardware modularity. IPMA also explained the utilization of cloud HIS. Even though hardware modularity is a highly important construct in formulating the utilization of cloud HIS, its performance needs to be improved. Thus, managers or decision-makers in the health sector should focus more on highly important constructs and improve their performance to enhance the system adoption process.

VII. Study implications

By identifying multiple variables strongly associated with the use of cloud health systems, the proposed model is different from others previously designed for use and adoption in the healthcare sector through the proposed variables, which are not combined in previous models with the adapted theories. Based on Rogers's (2003) [99] study, innovation adoption is not mature. He pointed out that the vast majority of studies in this field of innovation diffusion focus only on the characteristics of the innovation, and the number of studies considering various aspects of the context is limited. Furthermore, the results show no anomalies in the findings as all results follow prior studies and expectations of the current situation in Iraqi hospitals. The model could also be used to investigate how other technology advancements are utilized in the healthcare industry; for instance, it can be used to investigate the utilization of telemedicine, the Internet of Things, or any technological innovation within the healthcare sector in Iraq and the Middle East region that considers near environmental settings, especially the Gulf region. As such, this study fills a gap in the literature and advances the field of study.

Practically, this research can help scholars, healthcare practitioners, and decision-makers. Cloud providers must understand some essential factors that are not associated with the

technology but affect utilization decisions. Moreover, cloud providers may need to enhance their interactions with hospitals engaged in the cloud experience to create a suitable environment for utilizing cloud computing and eliminate any vagueness surrounding this particular type of innovative technology. Consequently, this research also guides cloud computing diffusion by providing the staff with the opportunity to improve their cloud usage by studying their behavior which will certainly influence the use of such services, thus offering benefits to the healthcare sector by focusing on the main antecedents that may be taken into consideration to facilitate the actual utilization process. Furthermore, the system developer(s) may consider these predictors throughout the development processes by understanding the main factors that could enhance the cloud utilization process.

VIII. Limitations and future works

This study has some limitations. For instance, the data collected were from the capital city of Iraq. Therefore, the findings might not be generalizable to other hospitals or countries. Additionally, the sample was limited to healthcare information technicians, who were chosen based on a simple random sampling technique. Moreover, the study utilized cross-sectional data. Although collecting longitudinal data may provide a strong generalization, this may also provide a measure for changes in respondents' attitudes over time due to the ongoing development of cloud computing technology. Furthermore, this study examined limited factors related to organizational structure, behavior, and technology utilization. Some other important antecedents may be considered in future research, such as security and privacy of health information (which many research consider technical variables). Thus, to start addressing these limitations, it is suggested that future researches take into account exploring other organizational, cultural, and technical predictors by expanding the proposed study model to support decision-makers in enhancing their healthcare systems and best utilization of technology to ensure more organized health records for better healthcare services.

IX. Conclusion

The utilization of trend technologies was raised dramatically. The researchers in the present study were motivated to investigate the potential role of cloud computing service utilization in the healthcare industry. Organizational factors are important to investigate regarding healthcare information technicians' behaviors. They are considered the main essential part of the adoption process. As such, a distinctive model has been proposed for utilizing a cloud health system in the context of Iraq. Hardware and software modularity, good internet quality, and a high level of training were found to positively influence healthcare information technicians' behavioral confirmation and control to utilize cloud technology in their workplace. As the theory of reasoned action suggests, individuals' behavioral changes construct normative beliefs and motivation to comply with the offered services. We found that changing individuals' confirmation regarding the offered technology will positively influence their usage behavior of cloud HISs. To further improve the functionality and utility of the system, developers must involve information technicians during the development and implementation phases. In Iraqi healthcare, these procedures will undoubtedly contribute to maintaining effective communication among healthcare professionals and ensuring the continuity of patient treatment. In order to get significantly better adoption in practice, hospital administration must allocate resources, provide training, and ensure user participation before adopting the system.

Financial disclosure

Publication was Funded by Universiti Kebangsaan Malaysia, under the grant DPB-2020-308.

Supporting information

S1 File.
(DOCX)

S1 Data.
(CSV)

Author Contributions

Conceptualization: Ahmed Meri.

Data curation: Ahmed Meri, Mohammad Khatim Hasan.

Formal analysis: Ahmed Meri, Mohammed Al-Bsheish.

Funding acquisition: Ali Aldujaili.

Investigation: Mohammad Khatim Hasan, Ali Aldujaili, Haitham Mohsin Kareem.

Methodology: Ahmed Meri, Mohammad Khatim Hasan, Mohammed Al-Bsheish.

Project administration: Mohammad Khatim Hasan.

Resources: Ahmed Meri, Mohammed Dauwed, Ali Aldujaili, Salah Shehab, Haitham Mohsin Kareem.

Software: Mohammed Dauwed, Salah Shehab.

Supervision: Mohammad Khatim Hasan, Mu'taman Jarrar.

Validation: Mohammed Dauwed, Mu'taman Jarrar, Haitham Mohsin Kareem.

Visualization: Mohammed Dauwed, Mu'taman Jarrar, Haitham Mohsin Kareem.

Writing – original draft: Ahmed Meri, Mohammad Khatim Hasan.

Writing – review & editing: Mohammad Khatim Hasan, Mohammed Dauwed, Mu'taman Jarrar, Ali Aldujaili, Mohammed Al-Bsheish, Salah Shehab.

References

1. Hussain AA, Bouachir O, Al-Turjman F, Aloqaily M. AI Techniques for COVID-19. IEEE Access. 2020; 8:128776–95. https://doi.org/10.1109/ACCESS.2020.3007939 PMID: 34976554
2. Cheng Y-M. Quality antecedents and performance outcome of cloud-based hospital information system continuance intention. J Enterp Inf Manag. 2020;
3. Georgiopoulou Z, Makri E-L, Lambrinoudakis C. GDPR compliance: proposed technical and organizational measures for cloud provider. Inf Comput Secur. 2020;
4. Dahri AS, Thebo LA. An overview of AI enabled M-IoT wearable technology and its effects on the conduct of medical professionals in Public Healthcare in Pakistan. 2020;
5. Celesti A, Ruggeri A, Fazio M, Galletta A, Villari M, Romano A. Blockchain-Based Healthcare Workflow for Tele-Medical Laboratory in Federated Hospital IoT Clouds. Sensors. 2020; 20(9):2590. https://doi.org/10.3390/s20092590 PMID: 32370129
6. Yusof MM, Aziz KABD. Evaluation of Organizational Readiness in Information Systems Adoption: A Case Study. Asia-Pacific J Inf Technol Multimed. 2015; 4(2):69–86.
7. Mukred M, Yusof ZM, Noor NABM, Kayode BK, Al-Duais R. The Role of Cloud Electronic Records Management System (ERMS) Technology in the Competency of Educational Institutions. In: International Conference of Reliable Information and Communication Technology. Springer; 2019. p. 936–46.
8. Wang X, Tang Y, Song R, Li J. A new model for investigating the effective factors in the development of modern clinical and health services in the time of COVID-19. Inf Syst E-bus Manag. 2023;1–25.

9. Rosati F, Faria LGD. Business contribution to the Sustainable Development Agenda: Organizational factors related to early adoption of SDG reporting. Corp Soc Responsib Environ Manag. 2019; 26 (3):588–97.
10. Al Hilfi TK, Lafta R, Burnham G. Health services in Iraq. Lancet [Internet]. 2013; 381(9870):939–48. Available from: https://doi.org/10.1016/S0140-6736(13)60320-7 PMID: 23499042
11. Cetorelli V, Shabila NP. Expansion of health facilities in Iraq a decade after the US-led invasion, 2003–2012. Confl Health. 2014; 8(1):16. https://doi.org/10.1186/1752-1505-8-16 PMID: 25221620
12. Hameed RT, Mohamad OA, Hamid OT, Tapus N. Design of e-Healthcare Management System Based on Cloud and Service Oriented Architecture. 5th IEEE Int Conf E-Health Bioeng—EHB 2015. 2015;1–4.
13. Olakotan OO, Yusof MM. Evaluating the alert appropriateness of clinical decision support systems in supporting clinical workflow. J Biomed Inform. 2020;103453. https://doi.org/10.1016/j.jbi.2020.103453 PMID: 32417444
14. Selvaraj S, Sundaravaradhan S. Challenges and opportunities in IoT healthcare systems: a systematic review. SN Appl Sci. 2020; 2(1):139.
15. Zhai Y, Wang Y, Zhang M, Gittell JH, Jiang S, Chen B, et al. From Isolation to Coordination: How Can Telemedicine Help Combat the COVID-19 Outbreak? medRxiv. 2020;
16. Al-Hashimy HNH, Hamoud AK, Hussain FA. The Effect of Not Using Internet of Things in Critical life Situations in the Health Field and the Effect on Iraqi Profitability: Empirical Study in Basra. J Southwest Jiaotong Univ. 2019; 54(4).
17. Kadhum AM, Hasan MK. Assessing the Determinants of Cloud Computing Services for Utilizing Health Information Systems: A Case Study. Int J Adv Sci Eng Inf Technol. 2017; 7(2):503–10.
18. Hatch MJ, Cunliffe AL. Organization theory: modern, symbolic and postmodern perspectives. Third edit. Vol. 53, Oxford university press. Oxford university press; 2013. 351 p.
19. Ajzen I, Fishbein M. Understanding attitudes and predicting social behaviour. Englewood Cliffs, NJ: Prentice-Hall; 1980.
20. Gao F, Sunyaev A. Context matters: A review of the determinant factors in the decision to adopt cloud computing in healthcare. Int J Inf Manage. 2019; 48:120–38.
21. Madden TJ, Ellen PS, Ajzen I. A comparison of the theory of planned behavior and the theory of reasoned action. Personal Soc Psychol Bull. 1992; 18(1):3–9.
22. Ajzen I, Fishbein M. Attitudes and normative beliefs as factors influencing behavioral intentions. J Pers Soc Psychol. 1972; 21(1):1.
23. Finlay KA, Trafimow D, Moroi E. The importance of subjective norms on intentions to perform health behaviors. J Appl Soc Psychol. 1999; 29(11):2381–93.
24. Jarrar M, Rahman HA, Minai MS, AbuMadini MS, Larbi M. The function of patient-centered care in mitigating the effect of nursing shortage on the outcomes of care. Int J Health Plann Manage. 2018; 33(2): e464–73. https://doi.org/10.1002/hpm.2491 PMID: 29380909
25. Wang X, Lee KM. The paradox of technology innovativeness and risk perceptions–A profile of Asian smartphone users. Telemat Informatics. 2020;101415.
26. Buchanan DA, Huczynski AA. Organizational behaviour. Pearson UK; 2019.
27. Jena A, Philipson T, Tomas P. Endogenous cost-effectiveness analysis in health care technology adoption. Vol. 07281, National Bureau of Economic Research. 2009. Report No.: w15032.
28. Philipson TJ, Jena AB. Endogenous cost-effectiveness analysis and health care technology adoption. J Health Econ. 2013; 32(1):172–80. https://doi.org/10.1016/j.jhealeco.2012.10.002 PMID: 23202262
29. Ling DI, Lynd LD, Harrison M, Anis AH, Bansback N. Early cost–effectiveness modeling for better decisions in public research investment of personalized medicine technologies. J Comp Eff Res. 2019; 8 (1):7–19. https://doi.org/10.2217/cer-2018-0033 PMID: 30525982
30. Griebel L, Prokosch H-U, Köpcke F, Toddenroth D, Christoph J, Leb I, et al. A scoping review of cloud computing in healthcare. BMC Med Inform Decis Mak [Internet]. 2015; 15(17):17. Available from: http://www.pubmedcentral.nih.gov/articlerender.fcgi?artid=4372226&tool=pmcentrez&rendertype=abstract https://doi.org/10.1186/s12911-015-0145-7 PMID: 25888747
31. Byrd TA, Douglas ET. An exploratory analysis of the value of the skills of IT personnel: Their relationship to IS infrastructure and competitive advantage. Decis Sci. 2001; 32(1):21–54.
32. Elhadi M, Sharif M. Learning Readiness Assessment Framework In Promoting E-Health Engagement Among Libyan Nurses. Universiti Sains Malaysia; 2014.
33. Hsu CL, Lin JCC. What drives purchase intention for paid mobile apps?-An expectation confirmation model with perceived value. Electron Commer Res Appl [Internet]. 2015; 14(1):46–57. Available from: http://dx.doi.org/10.1016/j.elerap.2014.11.003

34. Susanto A, Chang Y, Ha Y. Determinants of continuance intention to use the smartphone banking services: An extension to the expectation-confirmation model. Ind Manag Data Syst. 2016; 116(3):508–25.

35. Hajli N, Lin X. Exploring the Security of Information Sharing on Social Networking Sites: The Role of Perceived Control of Information. J Bus Ethics. 2016; 133(1):111–23.

36. Dadhich P. Security of Healthcare Systems with Smart Health Records Using Cloud Technology. In: Machine Learning with Health Care Perspective. Springer; 2020. p. 183–98.

37. Sant'Anna C, Figueiredo E, Garcia A, Lucena CJ. On the modularity of software architectures: A concern-driven measurement framework. In: In European Conference on Software Architecture [Internet]. Springer Berlin Heidelberg; 2007. p. 207–24. Available from: http://www.springerlink.com/index/P867367173685035.pdf

38. Akbar MA, Sang J, Khan AA, Shafiq M, Hussain S, Hu H, et al. Improving the quality of software development process by introducing a new methodology–AZ-model. IEEE Access. 2017; 6:4811–23.

39. Sun H, Ha W, Xie M, Huang J. Modularity's impact on the quality and productivity of embedded software development: A case study in a Hong Kong company. Total Qual Manag [Internet]. 2015; 26(11):1188–201. Available from: http://www.tandfonline.com/doi/abs/10.1080/14783363.2014.920179

40. Green GC, Collins RW, Hevner AR. Perceived control and the diffusion of software process innovations. J High Technol Manag Res. 2004; 15(1):123–44.

41. Steinbart PJ, Nath R. Problems and issues in the management of international data communications networks: the experiences of American companies. MIS Q. 1992; 16(1):55–76.

42. Tshimula JM, Togashi A, Ayuni NS. Towards Financial Inclusion-based Monetization Model for Startups Drive. In: 2018 IEEE Technology and Engineering Management Conference (TEMSCON). IEEE; 2018. p. 1–4.

43. Gibreel O, AlOtaibi DA, Altmann J. Social commerce development in emerging markets. Electron Commer Res Appl. 2018; 27:152–62.

44. Hossain A, Quaresma R, Rahman H. Investigating factors influencing the physicians' adoption of electronic health record (EHR) in healthcare system of Bangladesh: An empirical study. Int J Inf Manage. 2019; 44:76–87.

45. Petersone M, Krastins AV, Ketners K. In-Service Training System Organization Improvement at Customs Administrations BT—Entrepreneurship, Business and Economics—Vol. 1. In: Bilgin MH, Danis H, editors. Cham: Springer International Publishing; 2016. p. 201–16.

46. Ghaleb EAA, Dominic PDD, Fati SM, Muneer A, Ali RF. The assessment of big data adoption readiness with a technology–organization–environment framework: a perspective towards healthcare employees. Sustainability. 2021; 13(15):8379.

47. Bello IS, Arogundade FA, Sanusi AA, Ezeoma IT, Abioye-Kuteyi EA, Akinsola A. Knowledge and utilization of Information Technology among health care professionals and students in Ile-Ife, Nigeria: a case study of a university teaching hospital. J Med Internet Res. 2004; 6(4):e45. https://doi.org/10.2196/jmir.6.4.e45 PMID: 15631969

48. Tsai J-M, Cheng M-J, Tsai H-H, Hung S-W, Chen Y-L. Acceptance and resistance of telehealth: The perspective of dual-factor concepts in technology adoption. Int J Inf Manage. 2019; 49:34–44.

49. Lee MC. Explaining and predicting users' continuance intention toward e-learning: An extension of the expectation-confirmation model. Comput Educ [Internet]. 2010; 54(2):506–16. Available from: https://doi.org/http%3A//dx.doi.org/10.1016/j.compedu.2009.09.002

50. Ismail MB, Yusof ZM. The Impact of Individual Factors on Knowledge Sharing Quality. J Organ Knowl Manag. 2010; 13:1–12.

51. Zhang M, Luo M, Nie R, Zhang Y. Technical attributes, health attribute, consumer attributes and their roles in adoption intention of healthcare wearable technology. Int J Med Inform. 2017; 108:97–109. https://doi.org/10.1016/j.ijmedinf.2017.09.016 PMID: 29132639

52. Bhattacherjee A. Understanding information systems continuance: an expectation-confirmation model. MIS Q [Internet]. 2001; 25(3):351–70. Available from: http://highered.mcgraw-hill.com/sites/dl/free/0070968292/815271/smi68292_app9A.pdf

53. Khayer A, Bao Y, Nguyen B. Understanding cloud computing success and its impact on firm performance: an integrated approach. Ind Manag Data Syst. 2020;

54. Lu H-H, Lin W-S, Raphael C, Wen M-J. A study investigating user adoptive behavior and the continuance intention to use mobile health applications during the COVID-19 pandemic era: Evidence from the telemedicine applications utilized in Indonesia. Asia Pacific Manag Rev. 2022;

55. Findik-Coşkunçay D, Alkiş N, Özkan-Yildirim S. A structural model for students' adoption of learning management systems: An empirical investigation in the higher education context. J Educ Technol Soc. 2018; 21(2):13–27.

56. Gupta A, Yousaf A, Mishra A. How pre-adoption expectancies shape post-adoption continuance intentions: An extended expectation-confirmation model. Int J Inf Manage. 2020; 52:102094.
57. Bandura A, Freeman WH, Lightsey R. Self-efficacy: The exercise of control. Springer; 1999.
58. Ajzen I, Madden TJ. Prediction of goal-directed behavior: Attitudes, intentions, and perceived behavioral control. J Exp Soc Psychol. 1986; 22(5):453–74.
59. Iskandar YHP, Subramaniam G, Majid MIA, Ariff AM, Rao GKL. Predicting healthcare professionals' intention to use poison information system in a Malaysian public hospital. Heal Inf Sci Syst. 2020; 8 (1):1–15.
60. Bhattacherjee A, Perols J, Sanford C. Information technology continuance: A theoretic extension and empirical test. J Comput Inf Syst. 2008; 49(1):17–26.
61. Neuman WL, Kreuger L. Social work research methods: Qualitative and quantitative approaches. Allyn and Bacon; 2003.
62. Zikmund WG. Business research methods 7th ed., Thomson/South-Western. Appendices; 2003.
63. Zammani M, Razali R. An Empirical Study of Information Security Management Success Factors. Int J Adv Sci Eng Inf Technol. 2016; 6(6):904–13.
64. Predmore SR. A Checkup on Careers in Health Technology. Tech Connect Educ Careers. 2005; 80 (v5):34–7.
65. Ratnam KA, Dominic PDD, Ramayah T. A structural equation modeling approach for the adoption of cloud computing to enhance the Malaysian healthcare sector systems-level quality improvement. J Med Syst. 2014; 38(8):82.
66. Harfoushi O, Akhorshaideh AH, Aqqad N, Al Janini M, Obiedat R. Factors affecting the intention of adopting cloud computing in Jordanian hospitals. Commun Netw. 2016; 8(02):88.
67. Alharbi F, Atkins A, Stanier C. Understanding the determinants of Cloud Computing adoption in Saudi healthcare organisations. Complex Intell Syst. 2016; 2(3):155–71.
68. Sekaran U, Bougie R. Research methods for business: A skill building approach. John Wiley & Sons; 2016.
69. Bagozzi RP, Youjae Y. Specification, evaluation, and interpretation of structural equation models. J Acad Mark Sci. 2012; 40(1):8–34.
70. Hair JF, Hult GTM, Ringle CM, Sarstedt M. A primer on partial least squares structural equation modeling (PLS-SEM). 2nd editio. SAGE Publications Inc. Sage Publications; 2016.
71. Chyung SY, Roberts K, Swanson I, Hankinson A. Evidence-based survey design: The use of a midpoint on the Likert scale. Perform Improv. 2017; 56(10):15–23.
72. Tehrani SR, Shirazi F. Factors influencing the adoption of cloud computing by small and medium size enterprises (SMEs). In: In International Conference on Human Interface and the Management of Information. Springer International Publishing.; 2014. p. 631–42.
73. Tallon PP. Inside the adaptive enterprise: an information technology capabilities perspective on business process agility. Inf Technol Manag. 2008; 9(1):21–36.
74. Igbaria M, Guimaraes T, Davis GB. Testing the determinants of microcomputer usage via a structural equation model. J Manag Inf Syst. 1995; 11(4):87–114.
75. Lin HF, Lee GG. Impact of organizational learning and knowledge management factors on e-business adoption. Manag Decis. 2005; 43(2):171–88.
76. Taylor S, Todd PA. Understanding information technology usage: A test of competing models. Inf Syst Res. 1995; 6(2):144–76.
77. Davis FD. Perceived usefulness, perceived ease of use, and user acceptance of information technology. MIS Q. 1989; 13(3):319–40.
78. Jamieson S. Likert scales: how to (ab) use them. Med Educ. 2004; 38(12):1217–8. https://doi.org/10.1111/j.1365-2929.2004.02012.x PMID: 15566531
79. Ringle CM, Wende S, Becker J-M. SmartPLS 3. Boenningstedt SmartPLS GmbH, http//www.smartpls.com. 2015;
80. Henseler J, Ringle CM, Sarstedt M. A new criterion for assessing discriminant validity in variance-based structural equation modeling. J Acad Mark Sci. 2015; 43(1):115–35.
81. Henseler J, Ringle CM, Sinkovics RR. The use of partial least squares path modeling in international marketing. In: New challenges to international marketing. Emerald Group Publishing Limited; 2009. p. 277–319.
82. Limayem M, Cheung CMK. Understanding information systems continuance: The case of Internet-based learning technologies. Inf Manag. 2008; 45(4):227–32.

83. Geisser S. A predictive approach to the random effect model. Biometrika. 1974; 61(1):101–7.

84. Stone M. Cross-validatory choice and assessment of statistical predictions. J R Stat Soc Ser B. 1974;111–47.

85. Tenenhaus M, Vinzi VE, Chatelin Y-M, Lauro C. PLS path modeling. Comput Stat Data Anal. 2005; 48 (1):159–205.

86. de la Torre-Díez I, López-Coronado M, Vaca C, Aguado JS, de Castro C. Cost-utility and cost-effectiveness studies of telemedicine, electronic, and mobile health systems in the literature: a systematic review. Telemed e-Health. 2015; 21(2):81–5. https://doi.org/10.1089/tmj.2014.0053 PMID: 25474190

87. Henderson C, Knapp M, Fernández J-L, Beecham J, Hirani SP, Beynon M, et al. Cost-effectiveness of telecare for people with social care needs: the Whole Systems Demonstrator cluster randomised trial. Age Ageing. 2014; 43(6):794–800. https://doi.org/10.1093/ageing/afu067 PMID: 24950690

88. Saleem JJ, Plew WR, Speir RC, Herout J, Wilck NR, Ryan DM, et al. Understanding barriers and facilitators to the use of Clinical Information Systems for intensive care units and Anesthesia Record Keeping: A rapid ethnography. Int J Med Inform. 2015; 84(7):500–11. https://doi.org/10.1016/j.ijmedinf.2015.03.006 PMID: 25843931

89. Ahmadi H, Rad MS, Nazari M, Nilashi M, Ibrahim O. Evaluating the factors affecting the implementation of hospital information system (HIS) using AHP method. Life Sci J. 2014; 11(3):202–7.

90. Sidek YH, Martins JT. Perceived critical success factors of electronic health record system implementation in a dental clinic context: An organisational management perspective. Int J Med Inform. 2017; 107:88–100. https://doi.org/10.1016/j.ijmedinf.2017.08.007 PMID: 29029696

91. La Iacona S, Militello C, Serbanati LD, Mastratisi MA, Ricci FL, Gilardi MC. Personal health system: A tool to support the patient empowerment. In: E-Health and Bioengineering Conference (EHB), 2015. IEEE; 2015. p. 1–6.

92. Al-Sakran HO. Framework architecture for improving healthcare information systems using agent technology. Int J Manag Inf Technol. 2015; 7(1):17.

93. Finley B, Boz E, Kilkki K, Manner J, Oulasvirta A, Hämmäinen H. Does network quality matter? A field study of mobile user satisfaction. Pervasive Mob Comput. 2017; 39:80–99.

94. Joukes E, Cornet R, Abu-Hanna A, de BRUIJNE M, de KEIZER N. End-user expectations during an electronic health record implementation: a case study in two academic hospitals. In: MIE. 2015. p. 501–5. PMID: 25991198

95. Nkuma-Udah KI, Chukwudebe GA, Ahaiwe J, Ejeta K, Ndubuka GI. Developing an Appropriate and Affordable Expert System for Medical Diagnosis (ESMD) in Developing Countries. In: World Congress on Medical Physics and Biomedical Engineering, June 7–12, 2015, Toronto, Canada. Springer; 2015. p. 1405–8.

96. Gerdtz MF, Daniel C, Dearie V, Prematunga R, Bamert M, Duxbury J. The outcome of a rapid training program on nurses' attitudes regarding the prevention of aggression in emergency departments: a multi-site evaluation. Int J Nurs Stud. 2013; 50(11):1434–45. https://doi.org/10.1016/j.ijnurstu.2013.01.007 PMID: 23433724

97. Ebardo RA, Celis NJ. Barriers to the Adoption of Electronic Medical Records in Select Philippine Hospitals: A Case Study Approach. In: Proceedings of the 2019 5th International Conference on Computing and Artificial Intelligence. 2019. p. 123–8.

98. Guo R, Berkshire SD, Fulton L V, Hermanson PM. Predicting intention to use evidence-based management among US healthcare administrators: application of the theory of planned behavior and structural equation modeling. Int J Healthc Manag. 2019; 12(1):25–32.

99. Rogers EM. Diffusion Of Innovations. 5th editio. The Free Press New York, NY; 2003. 576 p.
